# Supplementary material for: Identifying barriers and facilitators in HIV-indicator reporting for different health facility performances: A qualitative case study
Source: PLoS One. 2021 Feb 25;16(2):e0247525. doi: 10.1371/journal.pone.0247525 (PMC7906392; doi:10.1371/journal.pone.0247525)
Supplement: S1 Appendix — (DOCX) [file pone.0247525.s001.docx]

**S1Appendix: Interview Guide**

| **Social-demographic and interview logistics details** | **Date:**  **Start Time:**  **End Time:**  **Facility level:**  **Reporting tool: EMR or Paper**  **Age:**  **Gender:**  **Respondent Position:**  **No. Years in position:**  **Level of Education:**  **Background:** | |
| --- | --- | --- |
| **Concepts** | **Attributes Assessed** | **Interview questions** |
| **RHIS process** | -Routine reporting process (data collection and transmission)  -Existence of Standard Operating Procedure for data quality checks, training schedules etc.  -Feedback mechanisms | 1. Which MOH731 indicators are reported in this facility? 2. Who does reporting and how many people are involved? 3. Describe the process used to collect and report MOH731 indicators? 4. Have there been any changes that affected reporting? 5. What is the deadline for reporting? 6. Are deadlines adhered to? 7. Are there any repercussions for not adhering to deadlines? 8. What SOPs do you use to ensure timeliness and completeness? 9. Are data quality checks conducted?   If so,  What is the procedure for conducting data quality checks?  How often are data quality checks conducted?  How is completeness in reporting determined? |
| **Technical Determinants** | -Technology  -Reporting tools  -Fit between processes(tasks) and technology | 1. What is the role of EMR (if exists) in routine reporting process? 2. If technology exists: What is the ease or not of use of technology in reporting MOH731 indicators?   If no technology: How is reporting without EMR?   1. What is the ease of use of reporting forms? 2. Are systems available when needed? (internet availability) 3. What barriers do you face when using technology? |
| **Behavioral determinants** | -People capabilities  -Motivation  -Fit between individual and technology  -Fit between processes (tasks) and individual (Competence) | 1. What challenges do you face in reporting? 2. What motivates you in reporting 3. How often are you trained in conducting reporting process and use of technology if any |
| **Organizational determinants** | **-**Training schedules  -Supervision  -Availability of resources  -Availability of personnel | 1. Are there training schedules for employees? 2. Are resources used for reporting enough? 3. Is there shortage in personnel conducting routine reporting? 4. What mechanisms have been put in place to improve technical issues, personnel, SOPs? |
