# Supplementary material for: Identifying barriers and facilitators in HIV-indicator reporting for different health facility performances: A qualitative case study
Source: PLoS One. 2021 Feb 25;16(2):e0247525. doi: 10.1371/journal.pone.0247525 (PMC7906392; doi:10.1371/journal.pone.0247525)
Supplement: Attachment — Submitted filename: Review_PONE-D-20-39745.docx [file pone.0247525.s002.docx]

Thanks for the invitation to review this manuscript.

**Introduction**: This article provides a useful summary of barriers and facilitators in HIV-indicator reporting. The authors make a critical contribution to knowledge, especially now when most LMICs strive to improve data to guide decisions and health systems’ improvement. The need for the study is elaborated. However, a few minor observations need clarification:

1. In the data collection section (line 125 – 126), it is interesting that one of the methods employed to collect data for this manuscript was the informal direct observation. However, it is not clear what data was collected using this method as it is not reported in this manuscript
2. In the ethics section, the article states that privacy and confidentiality were ensured. However, it is not clear as to how was this done?
3. The reporting of quotes needs consistence. While some quotes indicate the interviewer number, others are silent. For example, line 459
4. The manuscript may need some general formatting, especially deleting empty lines in some places between paragraphs and quotes – see line 442, 532
5. Drawing from the results, especially that there are differences between county and partner employees in terms of acceptability of roles, how can LMICs build sustainable health information systems in the absence of partners?
6. The paper would also benefit from a thorough and close editorial review of grammar. For example,

- line 207 – is an incomplete phrase
- line 275 – change “contributed” to contributing
- line 295 – edit – facilities were EMRs where not implemented
- line 320 – edit – where aware of the reporting deadline
